# The puzzling Venusian polar atmospheric structure reproduced by a general circulation model

**DOI:** 10.1038/ncomms10398

**Published:** 2016-02-01

**Authors:** Hiroki Ando, Norihiko Sugimoto, Masahiro Takagi, Hiroki Kashimura, Takeshi Imamura, Yoshihisa Matsuda

**Affiliations:** 1 Institute of Space and Astronautical Science, Japan Aerospace Exploration Agency, Sagamihara, Kanagawa 252-0222, Japan; 2 Research and Education Center for Natural Sciences, Department of Physics, Keio University, Yokohama, Kanagawa 223-8521, Japan; 3 Faculty of Science, Kyoto Sangyo University, Kita-ku, Kyoto 603-8555, Japan; 4 Japan Agency for Marine-Earth Science and Technology, Yokohama, Kanagawa 236-0001, Japan; 5 Department of Astronomy and Earth Sciences, Tokyo Gakugei University, Koganei, Tokyo 184-8501, Japan

## Abstract

Unlike the polar vortices observed in the Earth, Mars and Titan atmospheres, the observed Venus polar vortex is warmer than the midlatitudes at cloud-top levels (∼65 km). This warm polar vortex is zonally surrounded by a cold latitude band located at ∼60° latitude, which is a unique feature called ‘cold collar' in the Venus atmosphere. Although these structures have been observed in numerous previous observations, the formation mechanism is still unknown. Here we perform numerical simulations of the Venus atmospheric circulation using a general circulation model, and succeed in reproducing these puzzling features in close agreement with the observations. The cold collar and warm polar region are attributed to the residual mean meridional circulation enhanced by the thermal tide. The present results strongly suggest that the thermal tide is crucial for the structure of the Venus upper polar atmosphere at and above cloud levels.

## Abstract (web summary)

Unlike some planets, the Venusian polar vortex is warmer than the mid-latitudes at cloud-top level, but the mechanism behind this is unknown. Here, the authors use a general circulation model and suggest the cold collar and warm polar regions are due to residual mean meridional circulation intensified by thermal tides.

## Graphical abstract

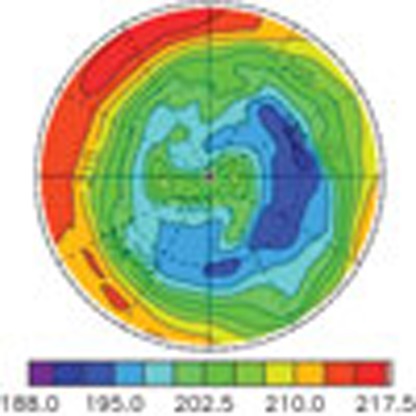

A lot of infrared and radio occultation measurements in the Venus exploration missions show that the polar vortex is warmer than the midlatitudes at cloud-top levels and surrounded by a zonal cold latitude band called the cold collar located around ∼60° latitude at ∼65 km (refs 1, 2, 3, 4). The morphology seen in the polar vortex changes temporally and sometimes shows a hot dipole or S-shaped structure^3^,^5^,^6^,^7^. Furthermore, these unique structures have been observed in both north and south polar regions.

While a lot of observational studies about the atmospheric structure in the Venus polar region have been performed, there were no numerical studies that succeeded in reproducing these unique structures in realistic model settings. Lee *et al*.^8^ have reported that the cold collar and the enhanced warm polar region (hot polar spot) are reproduced in their general circulation model (GCM) results and indicated that these structures are related to the reversal of the latitudinal temperature gradient, which is caused by the zonal jet produced by mean meridional circulation (MMC). However, the zonal-mean wind reproduced in their model is not consistent with recent measurements^9^. In addition, the MMC is driven by Newtonian cooling with constant relaxation time of 25 Earth days, in which diurnal variation of the solar heating is excluded. Yamamoto and Takahashi^10^,^11^ have also reproduced the polar vortex in their GCM with an artificial forcing of zonal wavenumber one at the bottom boundary to excite Kelvin and/or Rossby waves. Although they indicated that the polar diurnal tide enhances the cold collar and warm polar region, their results are not consistent with optical and radio occultation measurements for the following reasons: (1) the horizontal temperature distribution has large non-uniformity; (2) the location of the temperature minimum does not move in the longitudinal direction but is fixed at a specific geographical longitude; and (3) the temperature minimum corresponding to the cold collar is not seen in the latitude–height distribution of temperature in their calculation.

The Venus polar region also has interesting features in terms of material transport. Recent optical measurements showed that a significant infrared absorption signature by CO, which is mainly created photochemically above the cloud layer, exists over Venus' south pole, coinciding with bright dipole features seen in the polar vortex and the CO abundance increases with latitude above Venus' nightside cloud top^12^,^13^. Furthermore, the clouds in the polar region are thicker than those in the cold collar^14^. By using a two-dimensional model including a simple cloud microphysics scheme, Imamura and Hashimoto^15^ suggested that the clouds might become thick with latitude by the cloud material transport associated with the MMC. However, the structure of the MMC is simply assumed in their model. It is necessary to consider how its structure is related to the dynamical process.

A thermal tide is the planetary scale wave excited by the solar heating. In the Venus atmosphere, it is strongly excited at the cloud levels because a large part of the solar flux is absorbed there^16^,^17^,^18^. The horizontal wind velocity associated with the thermal tide is estimated to be >40 m s^−1^ at the cloud top. It is also indicated that superrotation at the cloud levels is affected significantly by the thermal tide^19^,^20^. However, its effect on the atmospheric structure in the polar region has not yet been examined.

In this study, we investigate the structure of the Venus upper polar atmosphere by using a GCM named Atmospheric GCM for the Earth Simulator (AFES) for Venus (see Methods for details). To examine the dynamical effects of the thermal tide, we perform two numerical experiments with observation-based distributions of the solar heating: one with the diurnal components (Case A) and one without them (Case B). The thermal tide is excited only in Case A. Our study shows that the cold collar and warm polar region are due to the residual mean meridional circulation (RMMC) intensified by the thermal tide, implying that the atmospheric structure at and above cloud levels in the Venus polar region is strongly attributed to the thermal tide. Furthermore, our results also indicate that the observed meridional distributions of cloud thickness and CO abundance above the cloud top are associated with the RMMC enhanced by the thermal tide.

## Results

### Background zonal wind and temperature distributions

The model atmosphere reached quasi-steady states within approximately four Earth years, and the quasi-steady states were maintained for more than ten Earth years^21^. In the following, we focus on the atmospheric structure in the northern hemisphere, because almost the same structure was observed in the southern hemisphere.

**Figure 1:**
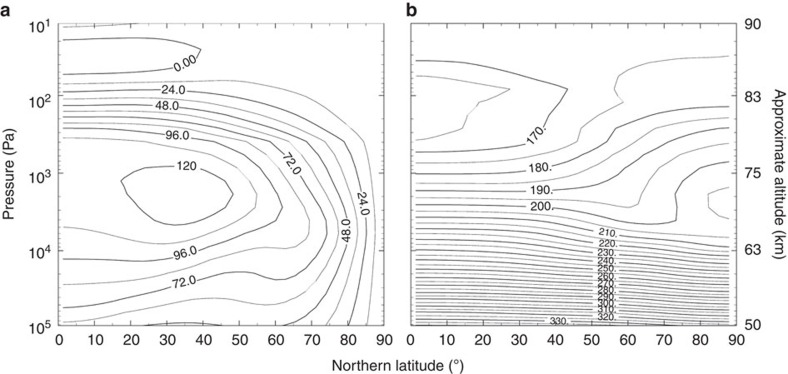
Meridional cross-sections of the zonally and temporally averaged data in Case A. (**a**) Zonal wind (m s^−1^) and (**b**) Temperature (K). Averaged period is two Venusian solar days (234 Earth days) after settling into the quasi-steady state.

Figure 1 shows meridional cross-sections of the zonally and temporally averaged zonal wind and temperature obtained in Case A. The fast mean zonal wind is maintained at 60–80 km. Above ∼80 km, the mean zonal wind decreases very sharply in the vertical direction; the velocity is <10 m s^−1^ at 90 km. In the temperature field, the meridional gradient changes its sign near the cloud-top level (∼70 km). That is, the zonal wind and temperature in our results are consistent with the gradient wind balance. As shown later, the warm polar region is related to the positive gradient of the temperature above that level. Significant negative shear of the mean zonal wind is attributed to the thermal tide, since it is theoretically predicted that the thermal tide accelerates the mean zonal wind at 50–70 km and decelerates at 80–100 km (ref. 20). Another notable feature is that the zonal wind velocity is almost unchanged vertically at 40–80 km levels in the polar region. These features of the zonal wind and the temperature are in close agreement with those obtained in recent observations^4^,^9^. Figure 1 also shows that a midlatitude jet is formed around the cloud-top level (∼70 km) at 20°–50° latitudes. The jet axis is located more equatorwards than that obtained in previous works^8^,^10^,^11^. This difference may also be explained by the acceleration effect of the thermal tide as noted above. The meridional distribution of the zonal wind is in close agreement with that reported by Machado *et al*.^22^, who retrieved the wind speed in the dayside by the ground-based Doppler velocimetry measurements. In the work of Piccialli *et al*.^9^, in which the meridional-height distributions of the zonal wind are estimated from those of the temperature by assuming the gradient wind balance, the positions of the midlatitude jets are about 15° polewards. It is noted, however, the estimated wind distribution strongly depends on the assumed meridional distribution of the zonal wind at the bottom boundary altitude. Sánchez-Lavega *et al*.^23^ also observed the wind distributions during the local time from 6 to 17 h, and indicated that the zonal wind speed increases from the morning to the afternoon due to the thermal tide at the upper cloud level in a latitude range of 50° S–75° S. This result is quite consistent with our present results. The zonal wind speed at middle and high latitudes is enhanced by the thermal tide by 20–30 m s^−1^ (not shown). It is also noted that, as shown in Sugimoto *et al*.^24^, the strength (or velocity) and the position of the midlatitude jet vary with time in the present model, although the variation width of the latter in the latitudinal direction is not more than ∼10°. These fluctuations may contribute to the difference between the present results and the observations. As shown later, the position of this jet is important to the cold collar.

### Temporal variation of temperature

**Figure 2:**
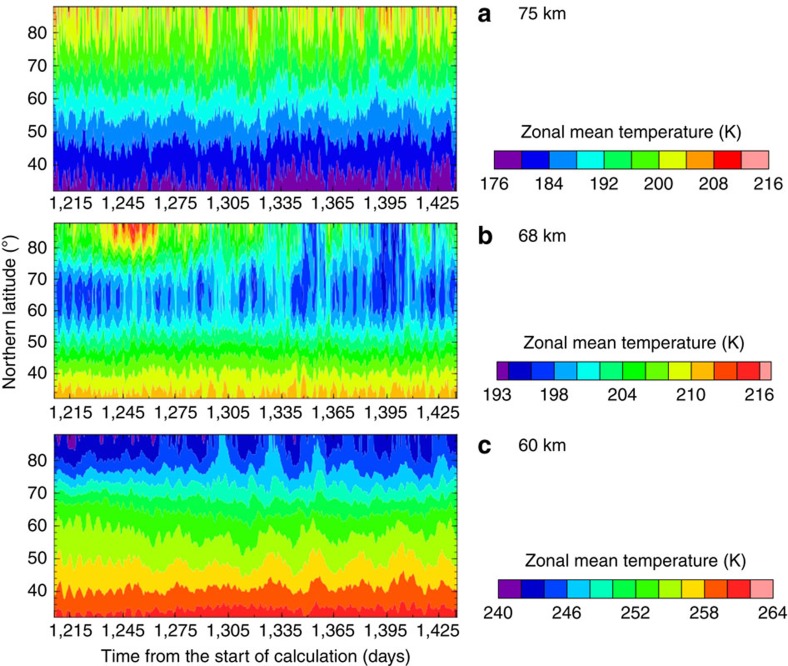
Time evolutions of the zonally averaged temperature in the quasi-steady state obtained in Case A. Each panel is depicted for two Venusian solar days (234 Earth days) at the altitude of (**a**) ∼75 km (the pressure level of 1 × 10^3^ Pa), (**b**) ∼68 km (4 × 10^3^ Pa) and (**c**) ∼60 km (2 × 10^4^ Pa), respectively. The latitude range is from 30° N to 90° N.

Figure 2 shows temporal variations of the zonal-mean temperature in a latitude range of 30°–90° at ∼75 km (the pressure level of 1 × 10^3^ Pa), ∼68 km (4 × 10^3^ Pa) and ∼60 km (2 × 10^4^ Pa) obtained in Case A. At ∼75 km, the polar region is always warmer than low latitudes with short- and long-period fluctuations (4–5 and ∼30 Earth days, respectively). The meridional temperature difference is ∼30 K, which is a little larger than that estimated from the radio occultation measurements^4^,^9^. At ∼68 km, the cold collar (local minimum of the temperature) clearly appears at latitudes of 60°–70°. The temperature in the cold collar is colder than the surroundings by ∼10 K, and qualitatively consistent with the radio occultation measurements^2^,^4^,^9^. The short-period fluctuation is dominant at this level, which may be caused by the baroclinic instability waves excited in the midlatitudes at the cloud levels^24^. At ∼60 km, the temperature monotonically decreases with latitude. Similarly to ∼75 km, fluctuations with both short and the long periods appear at this level.

**Figure 3:**
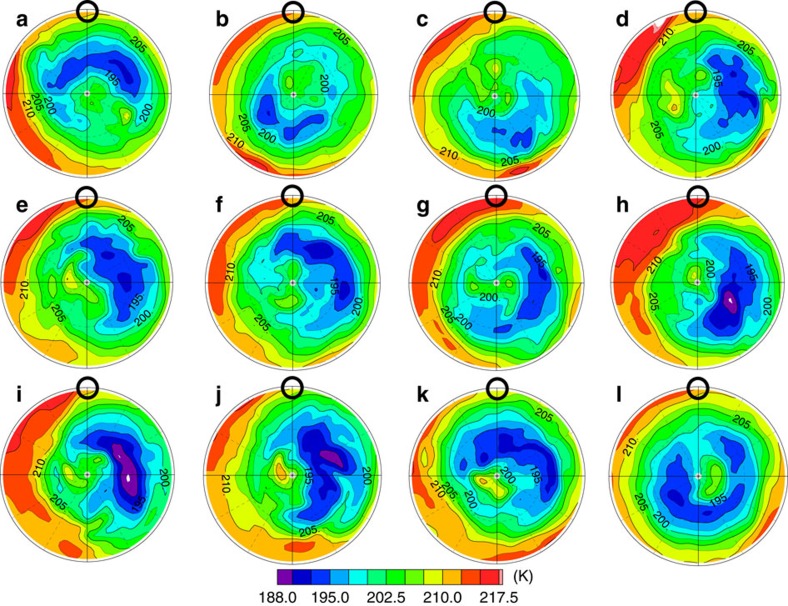
Time evolution of the temperatures (K) at∼68 km (the pressure level of 4 × 10^3^ Pa) in Case A in the polar plot. The latitude range is from 30°N to 90°N. The black circle represents the local solar noon. Each panel of (**a**) to (**l**) shows temperature distribution from 1414 to 1425 days from the start of the calculation. The time interval between the adjacent panels is one Earth day.

Figure 3 shows time evolution of the horizontal temperature distribution at ∼68 km obtained in Case A. The cold collar surrounds the warmer polar region at ∼60° N. The maximum temperature difference between 60° N and the pole is ∼20 K. The warm polar region rotates around the pole and varies in shape temporally. An S-shaped vortex structure also appears in Fig. 3g. It rotates around the pole, but the centre of rotation is offset from the pole. These features are in close agreement with the cold collar and the polar vortex observed by infrared measurements^1^,^3^,^5^,^6^, although the altitude of the cold collar reproduced in the present model is slightly higher than that estimated in such observations.

### Morphology in the Venus polar region

**Figure 4:**
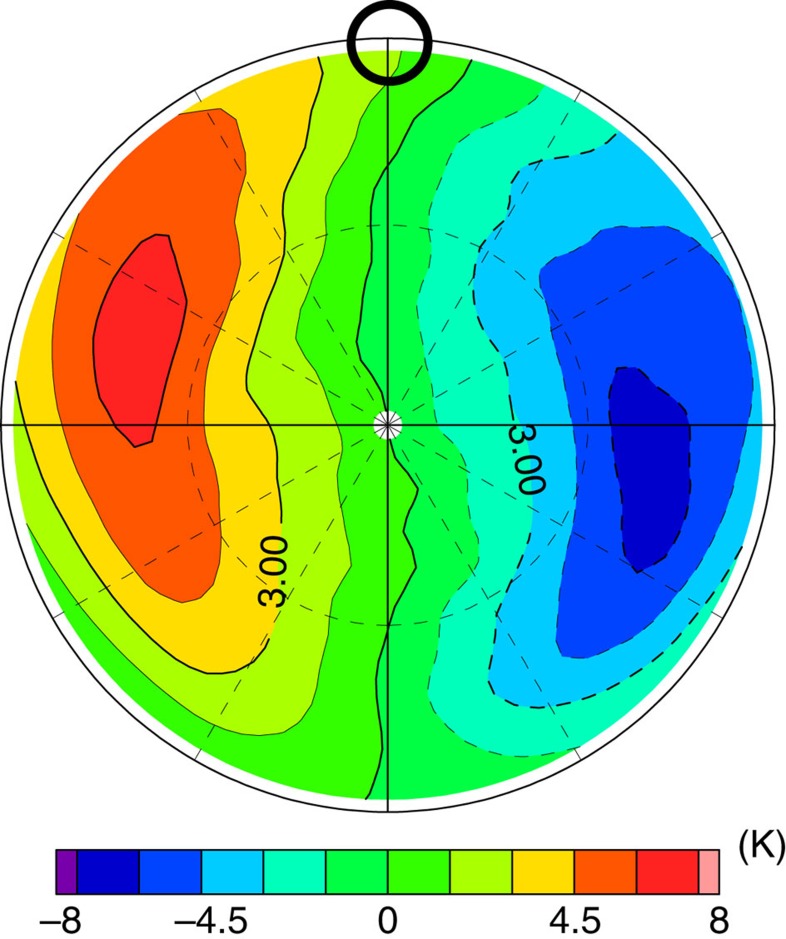
Polar plot of the temperature deviation (K) associated with the thermal tide at ∼68 km (the pressure level of 4 × 10^3^ Pa) obtained in Case A. The latitude range is from 30° N to 90° N. The black circle represents the local solar noon. Average period of data is 12 Earth days corresponding to the time interval for **a** to **l** in Fig. 3.

**Figure 5:**
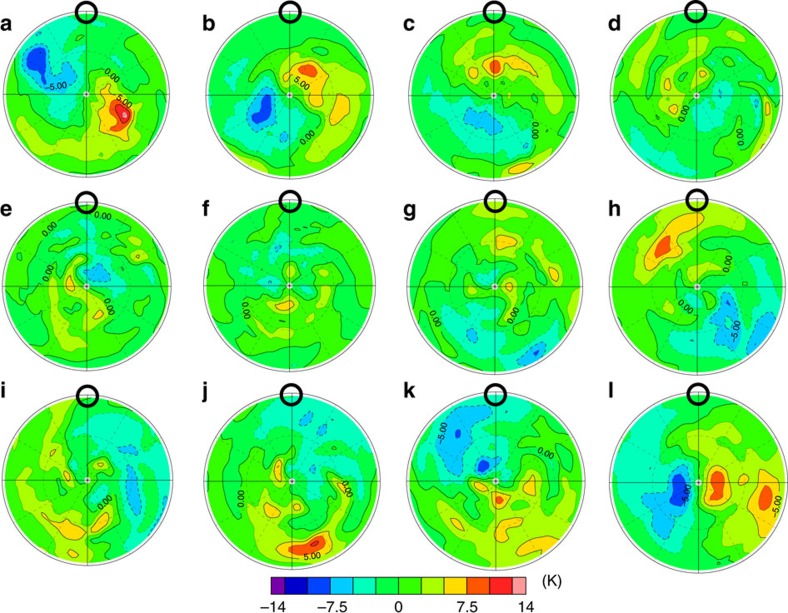
Polar plot of the temperature deviations (K) associated with the transient (short period) waves. The latitude range is from 30° N to 90° N. The black circle represents the local solar noon. Each panel of (**a**) to (**l**) shows temperature distribution from 1414 to 1425 days from the start of the calculation, corresponding to the time in Fig. 3. The time interval between the adjacent panels is one Earth day.

As shown in Fig. 3, zonal components with wavenumbers of zero, and one or two are predominant in the temperature distribution in the cold collar and the polar vortex. To elucidate the wave contributions, we extract the temperature components associated with the thermal tide and the short-period (transient) waves by using a frequency filter^25^, which are shown in Figs 4 and 5, respectively. Figures 3 and 4 indicate that the enhanced cold part in the cold collar is caused by the cold temperature phase of the thermal tide, which is consistent with Yamamoto and Takahashi^11^. The cold part of the cold collar is nearly always expanded into the morning side, and it sometimes surrounds the polar vortex completely (for example, Fig. 3b,g,k,l). Comparing Fig. 5 with Fig. 3, we found that this expansion is induced by the cold temperature phase of the transient waves. These results show that the cold collar is composed of the superposition of the zonally averaged basic field, the thermal tide and the transient waves.

It is also suggested from Figs 3 and 5 that the small-scale fluctuations seen in the polar vortex are mainly caused by the warm temperature phase of the transient waves. The morphology seen in the polar vortex is strongly affected by the transient waves, implying that the hot dipole or S-shaped structure observed by VIRTIS, the Visible and InfraRed Thermal Imaging Spectrometer on board Venus Express, would reflect the horizontal structure of the transient waves.

### Effect of thermal tide

**Figure 6:**
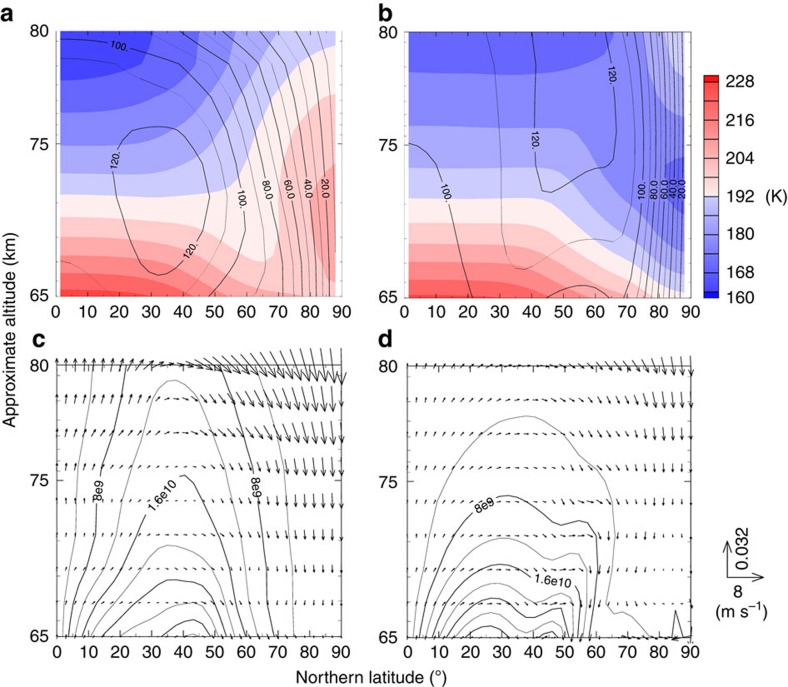
Meridional cross-sections of the zonally and temporally averaged zonal wind (solid line) and temperature (colour shade) and the horizontal and vertical components of the residual mean meridional circulation (vector) and mass stream fuction (contour). (**a**) Zonal wind and temperature in Case A. (**b**) Those in Case B. (**c**) Residual mean meridonal circulation vector and mass stream function in Case A. (**d**) Those in Case B. Averaged period is two Venusian solar days (234 Earth days) after settling into the quasi-steady state.

Figure 6a,b show latitude–height distributions of the zonally and temporally averaged zonal wind and temperature over two Venusian solar days (∼234 Earth days) obtained in Cases A and B. The axis of the midlatitude jet in Case A is located at a lower latitude and altitude than in Case B. This is likely due to the momentum transport by the thermal tide as mentioned above. In Case A, the temperature below 70 km altitude decreases with latitude up to 60° N in association with positive vertical shear of the mean zonal wind, whereas it increases with latitude poleward of 70° N. The remarkable cold collar is observed at 67–70 km levels at 60°–70° latitudes along with the polar warm region. In Case B, on the other hand, the temperature monotonically decreases with latitude at levels below 75 km height. Above 75 km, the temperature in the polar region is slightly warmer than in midlatitudes, but the temperature minimum corresponding to the cold collar is not reproduced, which is different from Case A.

RMMC defined by the transformed Eulerian-mean equations^26^ (Supplementary Note 1) is shown in Fig. 6c,d for Cases A and B, respectively. The RMMC represents the Lagrangian mean meridional circulation approximately. In Case A, the RMMC above the cloud-top level (∼70 km) reaches the polar region and remarkable downward motion exists there, which warms the atmosphere through adiabatic heating and forms the warm polar region. Also in Case B, the downward motion of the RMMC is observed in the polar region. However, it is 2–3 times slower than that in Case A (Supplementary Fig. 1). The adiabatic heating due to the downward motion in the polar region in Case B is much weaker than that in Case A.

**Figure 7:**
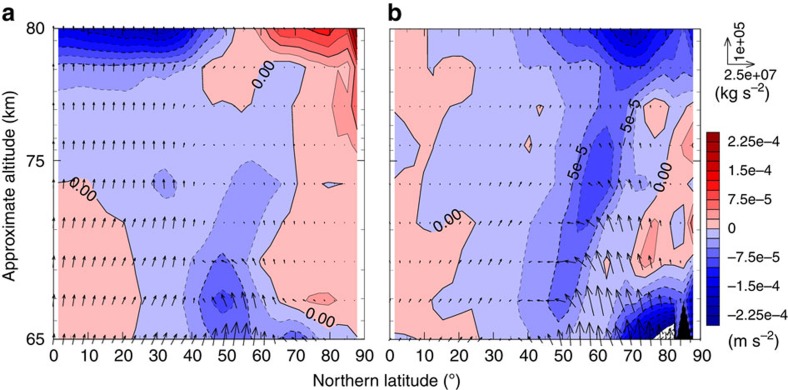
Meridional cross-sections of the EP flux (vectors) and its divergence (colours). (**a**,**b**) Cases A and B, respectively. Average period of data is two Venusian solar days (234 Earth days) after settling into the quasi-steady state.

These results imply that the thermal structure of Venus upper polar atmosphere (that is, the warm polar region and the cold collar) is attributed to the RMMC enhanced by the thermal tide. To investigate the effect of the thermal tide on the RMMC, we examined the momentum and heat balances in the transformed Eulerian-mean equations. The Eliassen–Palm (EP) flux and its divergence obtained for Cases A and B are shown in Fig. 7. In Case A, strong convergence of the EP flux (shown by blue shades) exists at latitudes equatorward of 55° N–60° N above 75 km, which is balanced mainly with upward advection of the negative vertical shear of the mean zonal wind due to the RMMC. On the other hand, the EP flux is divergent (shown by red shades) at latitudes poleward of 55° N–60° N. This divergence is balanced with the sum of poleward advection of the absolute vorticity and downward advection of the negative vertical shear of the mean zonal wind (Supplementary Fig. 2). Since these vertical motions of the RMMC induce adiabatic cooling and heating at the lower and higher latitudes, respectively, the clear structure of the cold collar has been reproduced in Case A.

In Case B, the EP flux is almost absent at lower latitudes as shown in Fig. 7b. Since the thermal tide is excluded in this case, it is inferred that the EP flux at the low latitudes obtained in Case A is induced by the thermal tide. Convergence of the EP flux is predominant at latitudes poleward of 20° N except at 68–77 km altitudes in the polar region, which is mainly balanced with poleward advection of the absolute vorticity due to the RMMC (Supplementary Fig. 2). The vertical advection can be neglected in Case B because both the vertical motions of the RMMC and the vertical shear of the mean zonal wind are much weaker than those obtained in Case A.

**Figure 8:**
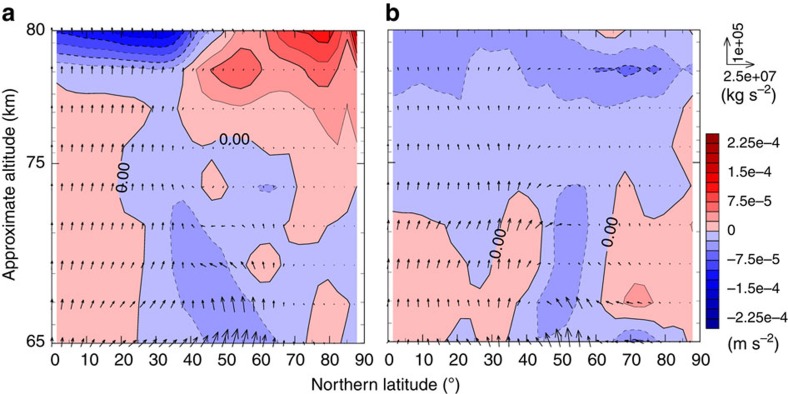
Meridional cross-sections of the EP flux (vectors) and its divergence (colour) in Case A. (**a**) Thermal tide and (**b**) Transient (short period) waves. Averaged period of data is two Venusian solar days (234 Earth days) after settling into the quasi-steady state.

Furthermore, to clarify the contribution of the thermal tide in Case A, we decompose the EP flux into long- and short-period components by using a frequency filter with a period of 10 Earth days, which correspond to the thermal tide and the transient waves, respectively. Figure 8 shows the EP flux divergence due to the thermal tide and the transient waves. Compared with Fig. 7a, a large part of the total EP flux divergence can be ascribed to the thermal tide, and the transient waves may not be neglected in the polar region below 70 km. These results indicate that the different dynamical states have been established in Cases A and B due to the thermal tide. In Case A, the RMMC is enhanced by the thermal tide, which induces the adiabatic polar warming, creating the cold collar (see Supplementary Note 2 and Supplementary Figs 3 and 4 for more details).

## Discussion

Our idea that the unique Venusian polar atmospheric structure is attributed to the RMMC enhanced by the thermal tide is different from Yamamoto and Takahashi^11^, who suggested that warm polar region is induced by the warm temperature phase of the diurnal tide. Imamura^27^ has pointed out that the RMMC above the cloud layer can be induced by upward propagating Rossby and/or gravity waves, but the thermal tide has not been taken into account in his study. Lebonnois *et al*.^28^ also performed GCM simulations, which includes more realistic physical parameterization schemes of the radiative transfer and boundary layer processes. They reported that the cold collar was not reproduced in their results, and inferred that the interaction between temperature and cloud structures, which was not included in their model, might be important for the cold collar formation. However, since the realistic cold collar has been reproduced in our model with the simplified radiative process, it is suggested that the cloud-radiative feedback is not essential for the cold collar. It should be also noted that an equatorial jet appeared in their results. This is different from our results, in which the jet is located at the midlatitudes. Because the temperature distribution is closely related with the mean zonal wind through the gradient wind balance, the realistic mean zonal wind and the RMMC enhanced by the thermal tide are crucial to the unique atmospheric structure seen in the Venus polar region.

In addition, we might be able to explain the recent infrared observational results about the meridional distributions of CO abundance and cloud thickness by taking into account the fact that the meridional and vertical motions of the RMMC shown in Fig. 6c approximately represent the material transport. It is inferred that the poleward material transport above the cloud top due to the RMMC leads to high CO abundance in the polar region, because on the way from the equator to the pole, an air parcel keeps gaining CO, which is created photochemically above the cloud layer, until it sinks in the polar region. Furthermore, H_2_SO_4_ liquid particles as well as CO are transported towards the polar region and grow in the same manner. The temperature at which H_2_SO_4_ liquid particles evaporate is almost equal to that at the cloud bottom altitude of ∼50 km (refs 15, 29). This temperature is much higher than that observed at altitudes above 65 km, where the RMMC transports the H_2_SO_4_ particles towards the poles in this study. Therefore, the clouds in the polar region are expected to become thicker than those in the cold collar. This material transport expected from the present result is qualitatively consistent with Imamura and Hashimoto^15^.

Our simulations elucidate the importance of the thermal tide for the Venus atmospheric circulation around the cloud-top level (∼70 km). The cold collar and the warm polar region, which are related to the zonal wind distribution through gradient wind balance, are realistically reproduced in the presence of the diurnal heating and can be explained by the downward motion of the RMMC enhanced by the thermal tide. This is qualitatively analogous to a sudden stratospheric warming in the Earth atmosphere, which is related to the meridional circulation induced by upward propagating Rossby waves^30^. In addition, the observed meridional distributions of cloud thickness and CO abundance above the cloud top are expected to be associated with the RMMC enhanced by the thermal tide. This is a new insight provided by the present work and is helpful for interpreting numerous observations and features of the Venus upper atmosphere.

Although the importance of the thermal tide is revealed in this study, further details of the mechanism how the structure of the RMMC is determined should be elucidated by investigating the wave propagation in our model and analysing observational data in the near future. In this December, the Venus Climate Orbiter ‘Akatsuki' will start observations^31^,^32^. Data provided by Akatsuki will also greatly help us to understand the real Venus atmosphere.

## Methods

### Set-up of AFES for Venus

AFES for Venus is a full nonlinear GCM on the sphere constructed for the Venus atmosphere^21^,^24^. The basic equations are primitive equations in sigma coordinates without topography. The physical parameter values applied in this study are as follows: planet radius is 6,050 km; gravity acceleration 8.87 m s^−2^; planetary rotation period 243 Earth days; and standard surface pressure 92 × 10^5^ Pa. The specific heat at constant pressure *C*_p_ is assumed to be constant at 1,000 J kg^−1^ K^−1^ for simplicity. It is noted that *C*_p_ varies approximately from 1.18 × 10^3^ to 7.38 × 10^2^ J kg^−1^ K^−1^ in the Venus atmosphere^33^. The resolution is set to T42L60 (128 × 64 horizontal grids and 60 vertical levels). The vertical domain extends from the ground to ∼120 km, with an almost constant grid spacing of 2 km. The model includes horizontal and vertical eddy diffusion. The former is represented by horizontal hyper-viscosity ∇^4^ and has a relaxation time for the largest wavenumber of 0.1 Earth days. The vertical diffusion coefficient is assumed to be constant at 0.15 m^2^ s^−1^. Rayleigh friction is employed at the lowest level to mimic surface friction. A sponge layer is used to damp eddy components of the wind above 80 km. However, its effect becomes dominant above 100 km because the damping coefficient is very small at 80–100 km levels.

### Radiative processes

Radiative transfer in the infrared region is represented simply by Newtonian cooling with the coefficients based on previous studies^20^,^34^. Furthermore, the temperature field is relaxed to the prescribed horizontally uniform distribution, whose vertical profile is based on the Venus International Reference Atmosphere. The period of the data analysis in this study is two Venusian solar days (234 Earth days) after settling into the quasi-steady state. The solar heating is based on previous studies^34^,^35^. In the present study, it is decomposed into a zonal-mean component and deviation from the zonal mean (diurnal component), which excites the mean meridional (Hadley) circulation and the thermal tide, respectively. Case A includes both the components, whereas Case B includes only the zonal mean. See Sugimoto *et al*.^21^,^24^ for further details of the model settings.

## Additional information

**How to cite this article:** Ando, H. *et al*. The puzzling Venusian polar atmospheric structure reproduced by a general circulation model. *Nat. Commun.* 7:10398 doi: 10.1038/ncomms10398 (2016).

## Supplementary Material

Supplementary Information Supplementary Figures 1-4, Supplementary Notes 1-2 and Supplementary Reference
