# Supplementary material for: The puzzling Venusian polar atmospheric structure reproduced by a general circulation model
Source: Nat Commun. 2016 Feb 1;7:10398. doi: 10.1038/ncomms10398 (PMC4740379; doi:10.1038/ncomms10398)
Supplement: Supplementary Information — Supplementary Figures 1-4, Supplementary Notes 1-2 and Supplementary Reference [file ncomms10398-s1.pdf]

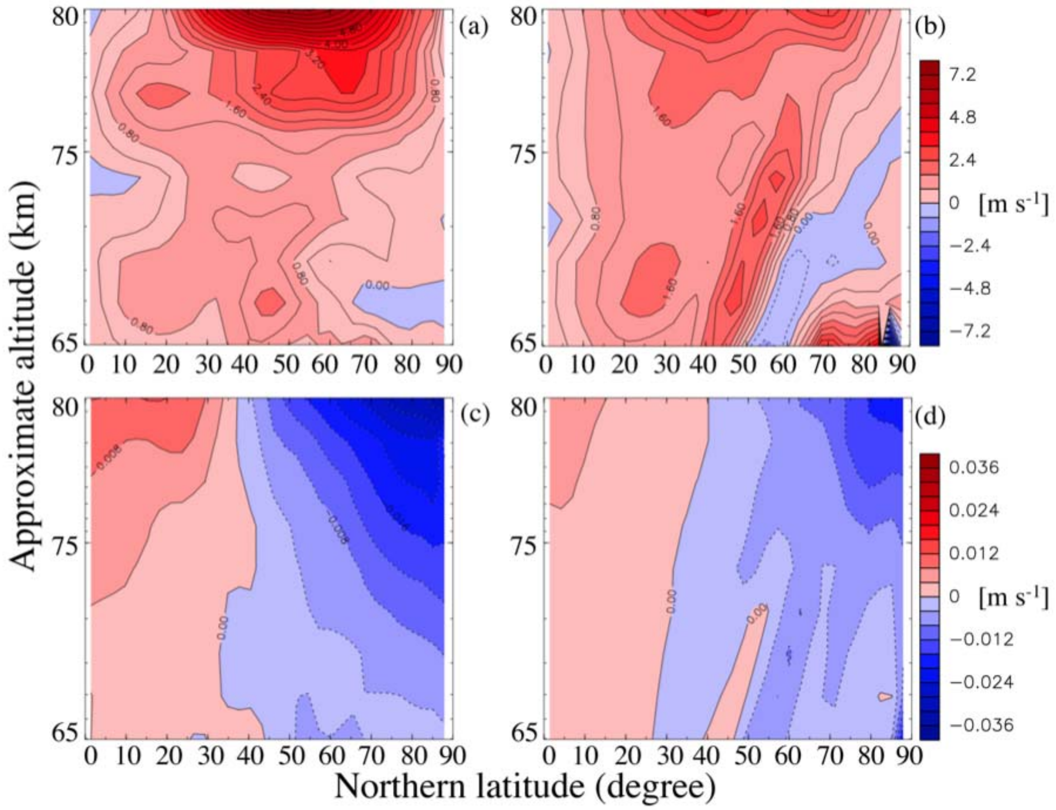

Supplementary Figure 1 | Meridional cross sections of the zonally and temporally averaged meridional (a and b) and vertical (c and d) components of the RMMC.

(a) and (**c)** are depicted for Case A, and (**b)** and (**d**) are for Case B. The data are averaged for two Venusian solar days (234 Earth days) in the quasi-steady state.

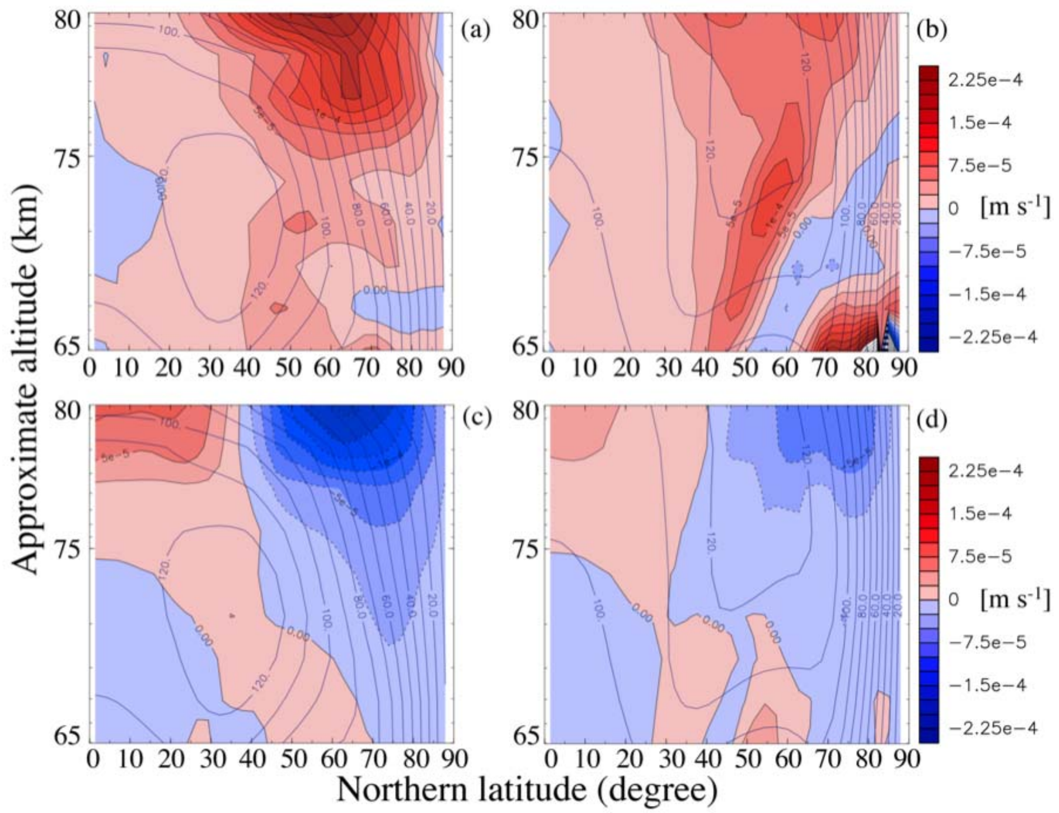

Supplementary Figure 2 | Meridional cross sections of the meridional (a and b) and vertical (c and d) momentum advections due to the RMMC. The zonally and temporally averaged mean zonal wind (blue solid lines) is superimposed. **(a)** and (**c**) are depicted for Case A, and (**b)** and (**d**) are for Case B. The data are averaged for two Venusian solar days (234 Earth days) in the quasi-steady state.

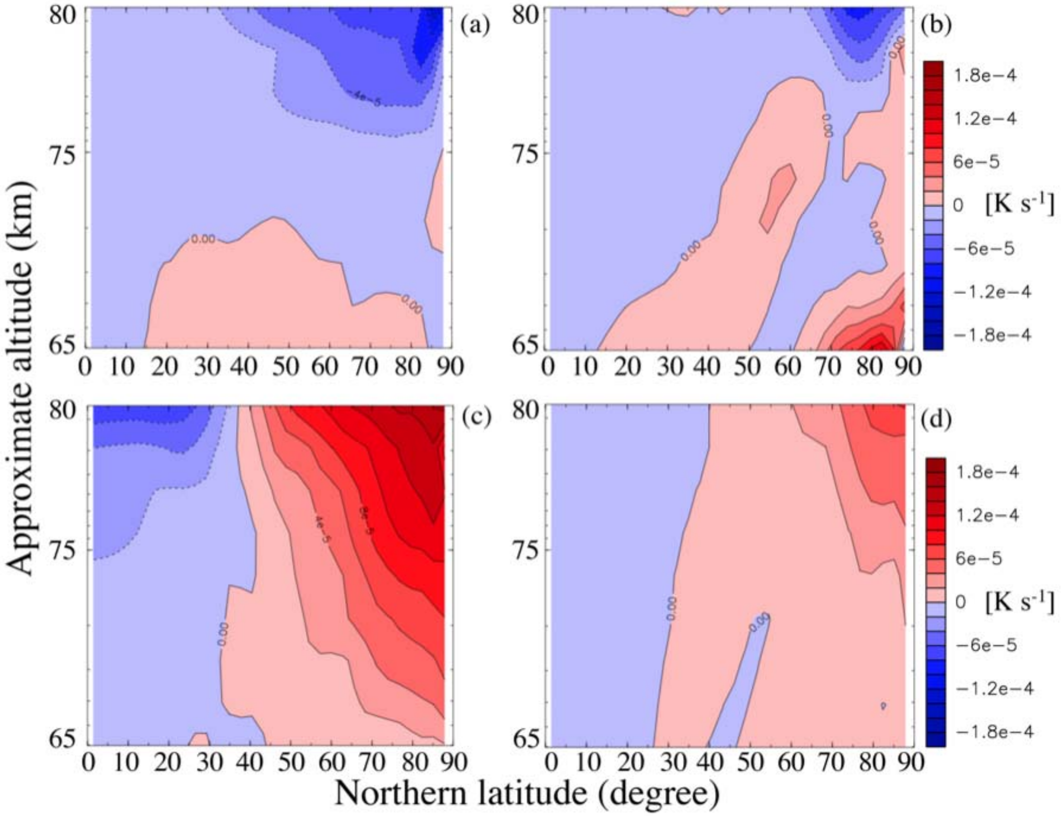

Supplementary Figure 3 | Meridional cross sections of the heating rates due to the meridional (a and b) and vertical (c and d) advections by the RMMC. (a) and (**c**) are depicted for Case A, and (**b)** and (**d**) are for Case B. The data are averaged for two Venusian solar days (234 Earth days) in the quasi-steady state.

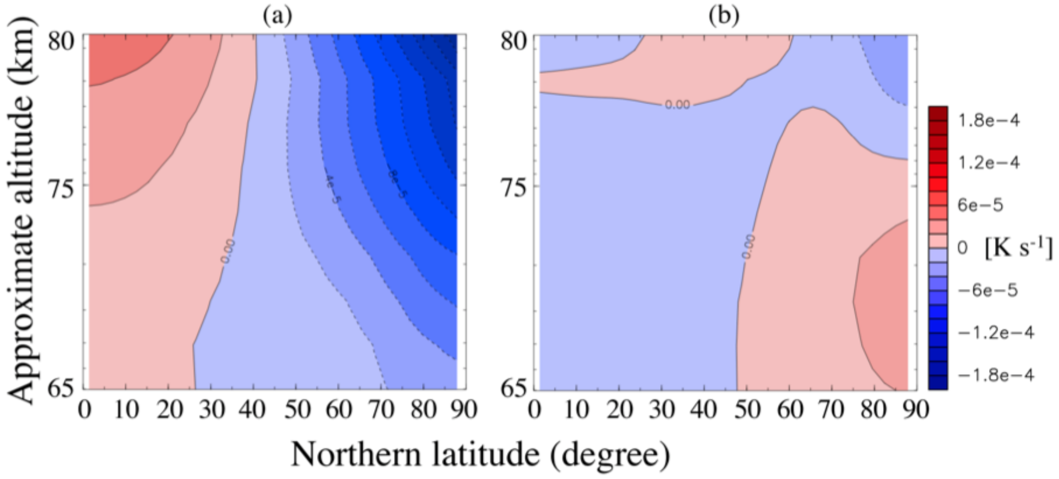

Supplementary Figure 4 | Meridional cross sections of the external heating rates, $\overline{Q}$, obtained for Cases A (a) and B (b). The heating rates are averaged for two Venusian solar days (234 Earth days) in the quasi-steady state.

Supplementary Note 1 Momentum balance in the TEM equations

Equation of zonal momentum in the Transformed Eulerian-Mean (TEM) equations in log-pressure coordinate are described as follows[1]:

$$\frac{\partial \overline{u}}{\partial t} = -\overline{v}^{*}\left[\frac{1}{a\cos\varphi}\frac{\partial}{\partial\varphi}\left(\overline{u}\cos\varphi\right) - f\right] - \overline{w}^{*}\frac{\partial \overline{u}}{\partial z} + \frac{\nabla \bullet \vec{\mathbf{F}}}{\rho_0 a\cos\varphi} + \overline{X}, \qquad (1)$$

where overbars denote zonal average, a is a planet radius, ρ_0 is atmospheric density, f is the Coriolis parameter, ϕ and z are latitude and log-pressure height, respectively. $\overline{v}^{*}$ and $\overline{w}^{*}$ are meridional and vertical components of the residual mean meridional circulation (RMMC) defined by

$$\overline{v}^{*} = \overline{v} - \frac{1}{\rho_0}\frac{\partial}{\partial z}\left(\frac{\rho_0 \overline{v'\theta'}}{\partial\overline{\theta}/\partial z}\right), \qquad (2)$$

$$\overline{w}^{*} = \overline{w} + \frac{1}{a\cos\phi}\frac{\partial}{\partial\phi}\left(\frac{\cos\phi\overline{v'\theta'}}{\partial\overline{\theta}/\partial z}\right). \qquad (3)$$

It is well-known that the RMMC represents the Lagrangian mean meridional circulation approximately. $\vec{\mathbf{F}}$ is the Eliassen-Palm flux (EP flux). The first and second terms in the right hand side (RHS) of Eq. (1) represent the meridional and vertical advections of zonal momentum by the RMMC. The third term including the divergence of the EP flux is the zonal acceleration due to wave activities. The last term ($\overline{X}$) represents external forcing due to viscosity and/or Rayleigh friction, which may be neglected below 100

km in the present study. It is noted that, since the left hand side (LHS) of Eq. (1) can be ignored in quasi-steady states, the terms in the RHS should be canceled out.

Supplementary Figure 1 shows meridional distributions of the horizontal and vertical components of RMMC, $\bar{v}^*$ and $\bar{w}^*$, in Cases A and B. In both the cases, upward and downward motions are predominant at lower and higher latitudes, respectively, and the meridional motion is poleward. Above 75 km, the meridional motion in Case A is 2–3 times as fast as that in Case B. As a result, the downward motion at higher latitudes in Case A is much stronger than that in Case B.

Supplementary Figure 2 shows the meridional cross sections of the meridional and vertical momentum advections due to the RMMC. These distributions obtained in Cases A and B are qualitatively similar to each other. However, as shown in Fig. 7, the distributions of the EP flux divergence are quite different in Cases A and B, implying that different dynamical balances have been established. In Case B, the meridional advection (Supplementary Figure 2 (b)) is almost balanced with the EP flux divergence (Fig. 7 (b)), and the vertical advection (Supplementary Figure 2 (d)) can be negligible except in narrow regions. In Case A, on the other hand, the meridional advection

(Supplementary Figure 2 (a)) is not balanced with the EP flux divergence (Fig. 7 (a)). Instead the sum of the meridional advection and the EP flux divergence is balanced with the vertical advection (Supplementary Figure 2 (c)). This result suggests that the vertical motions of the RMMC play important roles in the momentum balance in Case A. It is also indicated that, since the positive EP flux divergence in the polar region (Fig. 7 (a)) is canceled by the negative downward advection (Supplementary Figure 2 (c)), the downward motion at higher latitudes has been enhanced by the wave activities associated with the thermal tide (Fig. 8).

Supplementary Note 2 Heat balance in the TEM equations

Thermodynamic equation in the TEM equations in log-pressure coordinate is described as follows[1]:

$$\frac{\partial\bar{\theta}}{\partial t} = -\frac{\bar{v}^*}{a}\frac{\partial\bar{\theta}}{\partial\phi} - \bar{w}^*\frac{\partial\bar{\theta}}{\partial z} - \frac{1}{\rho_0}\frac{\partial}{\partial z}\left[\rho_0\left(\frac{\overline{v'\theta'}}{a}\frac{\partial\bar{\theta}/\partial\phi}{\partial\bar{\theta}/\partial z} + \overline{w'\theta'}\right)\right] + \bar{Q}, \quad (4)$$

where θ is potential temperature and Q is external diabatic heating. The first and second terms in the RHS of Eq. (4) represent the meridional and vertical heat advections by the RMMC. The third term heating due to wave activities. The last term ($\bar{Q}$) the radiative

transfer process including the solar heating and Newtonian cooling. As in Eq. (1), the terms in the RHS should be canceled out in quasi-steady states.

Supplementary Figure 3 shows meridional cross sections of the heating rates due to the horizontal and vertical heat advection by the RMMC in Cases A and B. Significant positive heating due to the downward motion exists at latitudes poleward of 40°N in both the cases. However, the magnitude of the polar heating in Case A is twice as large as in Case B. This difference is consistent with that of the RMMC, which has been enhanced by the thermal tide in Case A as discussed above. Supplementary Figure 4 shows the meridional cross sections of the external heating rates, $\overline{Q}$, for Cases A and B. It is confirmed in Case A that the strong adiabatic heating due to the downward motion of the RMMC is almost balanced with Newtonian cooling. This strong cooling is induced by the warm polar region (as shown in Figs. 1 and 6), which is formed by the strong adiabatic heating due to the downward motion of the RMMC. The present result indicates that the enhancement of the RMMC by the thermal tide induces the adiabatic polar warming, creating the cold collar and poleward temperature reversal at circumpolar latitudes.

Supplementary References

1. Andrews, D. G., Holton, J. R. & Leovy, C. B. Middle Atmosphere Dynamics, Academic Press, Orlando (1987).
